# Reproducibility of CT-based opportunistic vertebral volumetric bone mineral density measurements from an automated segmentation framework

**DOI:** 10.1186/s41747-024-00483-9

**Published:** 2024-08-01

**Authors:** Jannis Bodden, Philipp Prucker, Anjany Sekuboyina, Malek El Husseini, Katharina Grau, Sebastian Rühling, Egon Burian, Claus Zimmer, Thomas Baum, Jan S. Kirschke

**Affiliations:** 1 Department of Neuroradiology, TUM School of Medicine, Klinikum rechts der Isar, Technical University of Munich, Munich, Germany; 2 Department of Informatics, TUM School of Computation, Information and Technology, Technical University of Munich, Munich, Germany; 3 Department of Quantitative Biomedicine, University of Zurich, Zurich, Switzerland; 4 Department of diagnostic and interventional Radiology, University Hospital of Ulm, Ulm, Germany; 5 TUM-Neuroimaging Center, Klinikum rechts der Isar, Technical University of Munich, 81675 Munich, Germany

**Keywords:** Artificial intelligence, Bone density, Densitometry, Osteoporosis, Tomography (x-ray computed)

## Abstract

**Background:**

To investigate the reproducibility of automated volumetric bone mineral density (vBMD) measurements from routine thoracoabdominal computed tomography (CT) assessed with segmentations by a convolutional neural network and automated correction of contrast phases, on diverse scanners, with scanner-specific asynchronous or scanner-agnostic calibrations.

**Methods:**

We obtained 679 observations from 278 CT scans in 121 patients (77 males, 63.6%) studied from 04/2019 to 06/2020. Observations consisted of two vBMD measurements from *Δ*different *reconstruction kernels* (*n* = 169), *Δcontrast phases* (*n* = 133), *scan Δsessions* (*n* = 123), *Δscanners* (*n* = 63), or *Δall* of the aforementioned (*n* = 20), and observations *lacking scanner-specific calibration* (*n* = 171). Precision was assessed using root-mean-square error (RMSE) and root-mean-square coefficient of variation (RMSCV). Cross-measurement agreement was assessed using Bland-Altman plots; outliers within 95% confidence interval of the limits of agreement were reviewed.

**Results:**

Repeated measurements from *Δdifferent reconstruction kernels* were highly precise (RMSE 3.0 mg/cm^3^; RMSCV 1.3%), even for consecutive scans with different *Δcontrast phases* (RMSCV 2.9%). Measurements from different *Δscan sessions* or *Δscanners* showed decreased precision (RMSCV 4.7% and 4.9%, respectively). Plot-review identified 12 outliers from different *scan Δsessions*, with signs of hydropic decompensation. Observations with *Δall* differences showed decreased precision compared to those *lacking scanner-specific calibration* (RMSCV 5.9 and 3.7, respectively).

**Conclusion:**

Automatic vBMD assessment from routine CT is precise across varying setups, when calibrated appropriately. Low precision was found in patients with signs of new or worsening hydropic decompensation, what should be considered an exclusion criterion for both opportunistic and dedicated quantitative CT.

**Relevance statement:**

Automated CT-based vBMD measurements are precise in various scenarios, including cross-session and cross-scanner settings, and may therefore facilitate opportunistic screening for osteoporosis and surveillance of BMD in patients undergoing routine clinical CT scans.

**Key Points:**

- Artificial intelligence-based tools facilitate BMD measurements in routine clinical CT datasets.
- Automated BMD measurements are highly reproducible in various settings.
- Reliable, automated opportunistic osteoporosis diagnostics allow for large-scale application.

**Graphical Abstract:**

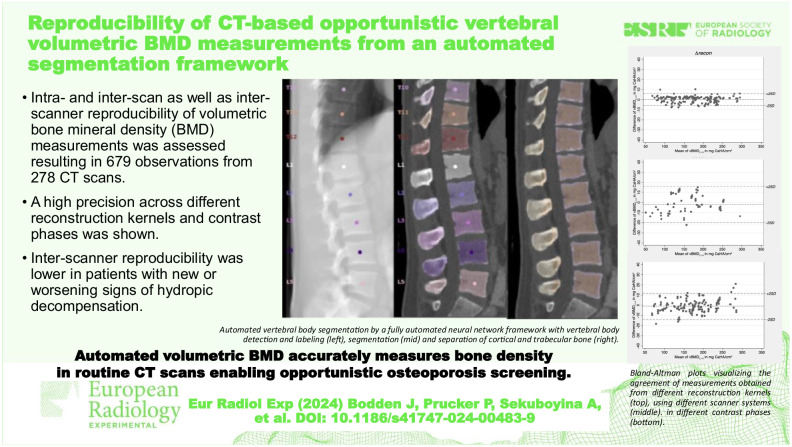

## Background

Osteoporosis is a systemic disease, that destabilizes the bone by demineralization of the osseous tissue and deterioration of the trabecular microstructure [1, 2]. The diagnosis is frequently delayed, because patients remain symptom free, until fragility fractures occur. Those occur in the absence of adequate trauma, but inherit significant morbidity, mortality and enormous socioeconomic consequences [3]. Demographic change aggravates this issue. In the USA, approximately 54 million people over the age of 50 were estimated to suffer from low bone mass or osteoporosis by 2010, and the number is projected to reach 71 million by 2030 [4].

Dual x-ray absorptiometry and quantitative computed tomography (CT) suited to screen for and diagnose osteoporosis are available [5, 6]. However, osteoporosis remains vastly underdiagnosed [2, 6, 7]. Part of the problem is that both methods rely on specialized tools (*e.g*., calibration phantoms), and, importantly, necessitate a dedicated exam, which inherits a substantial organizational effort.

Opportunistic approaches aim to overcome the limitations by determining bone mineral density (BMD) from exams performed for other indications [8]. The abundance of CT data underscores the possible impact of CT-based opportunistic BMD screening approaches: annual scans in the USA surpassed 278 per 1,000 inhabitants by 2019 [9]. However, the need for exact manual segmentations to determine trabecular volumetric BMD (vBMD) from routine clinical multidetector CT scans limited the viability of this approach so far.

Deep learning-based convolutional neural network frameworks have recently been developed to master this challenge [10]. Such neural networks automatically perform all steps of vertebral body segmentation. The conversion of asynchronous CT-based density values measured in HU into vBMD and corrections for the intravenous contrast media phase can be performed automatically [11–13]. However, to render this approach suitable for mass application, reproducibility has yet to be determined.

Thus, this study aimed to provide comprehensive information on the reproducibility of vBMD measurements performed by a fully automated convolutional neural network framework, utilizing routine clinical thoracoabdominal CT, obtained in different contrast media phases on a diverse set of scanner systems, with varying settings, asynchronously calibrated as well as using a manufacturer-generic, kVp-based calibration.

## Methods

### Study population

Patients who received at least two consecutive routine thoracoabdominal CT scans with an interscan interval of up to one month and matching regions of interest between April 2019 and June 2020 were identified from the local picture archiving and communication system. The maximum interscan interval of one month was selected to maximize the number of patients eligible for inclusion, while simultaneously aiming to rule out longitudinal changes in vBMD, which have been reported to reach 2% per year in a healthy cohort [14]. To cover the widest possible range of CT scanning systems in this study, scans performed at other institutions but that were imported into our system were also included. All scans were manually checked for lumbar spine coverage by a neuroradiology resident (P.P., 3 years of experience in spine imaging). Scans not covering the lumbar spine, as well as scans with severe beam hardening artifacts or high noise level at the lumbar spine (*e.g*., due to implants or other foreign material), were excluded. Further exclusion parameters were the presence of inflammatory and neoplastic lesions at the lumbar spine.

### Dataset acquisition, scanner calibration, and automated vertebral body segmentation and vBMD extraction

In-house contrast-enhanced scans were performed with a bodyweight-adjusted dosage (≤ 80 kg, 80 mL; 80–100 kg, 90 mL; > 100 kg, 100 mL) of iodined contrast media (Imeron 300, Bracco Imaging Deutschland GmbH, Konstanz, Germany). Tube voltage was 120 kVp (*n* = 176), or 100 kVp (*n* = 3) for in-house scanners, with an average tube load of 200 mAs.

In-house scans were obtained on a set of four scanners (Philips Brilliance iCT 256, Philips IQon Spectral CT, and Philips Ingenuity, Philips Medical Systems, Hamburg, Germany; Siemens Somatom Definition AS + , Siemens Healthineers, Erlangen, Germany). External scans were performed on one of eight scanner types (Canon Aquilion, and Canon Aquilion PRIME, Canon Medical Systems, Amstelveen, Netherlands; Siemens Biograph, Siemens Somatom Emotion 16, Siemens Somatom Definition AS, Siemens Somatom Force and Siemens Somatom Emotion 6, Siemens Healthineers, Erlangen, Germany; and Philips Ingenuity Core 128, Philips Medical Systems, Hamburg, Germany).

All reformations with a spatial resolution of ≤ 3 mm craniocaudally and of 5 mm left-right or anterior-posterior were included. In-house scanners were asynchronously calibrated using a commercially available anthropomorphic spine phantom (QRM QSA-717 Phantom; Quality Assurance in Radiology and Medicine GmbH, Möhrendorf, Germany).

**Fig. 1:**
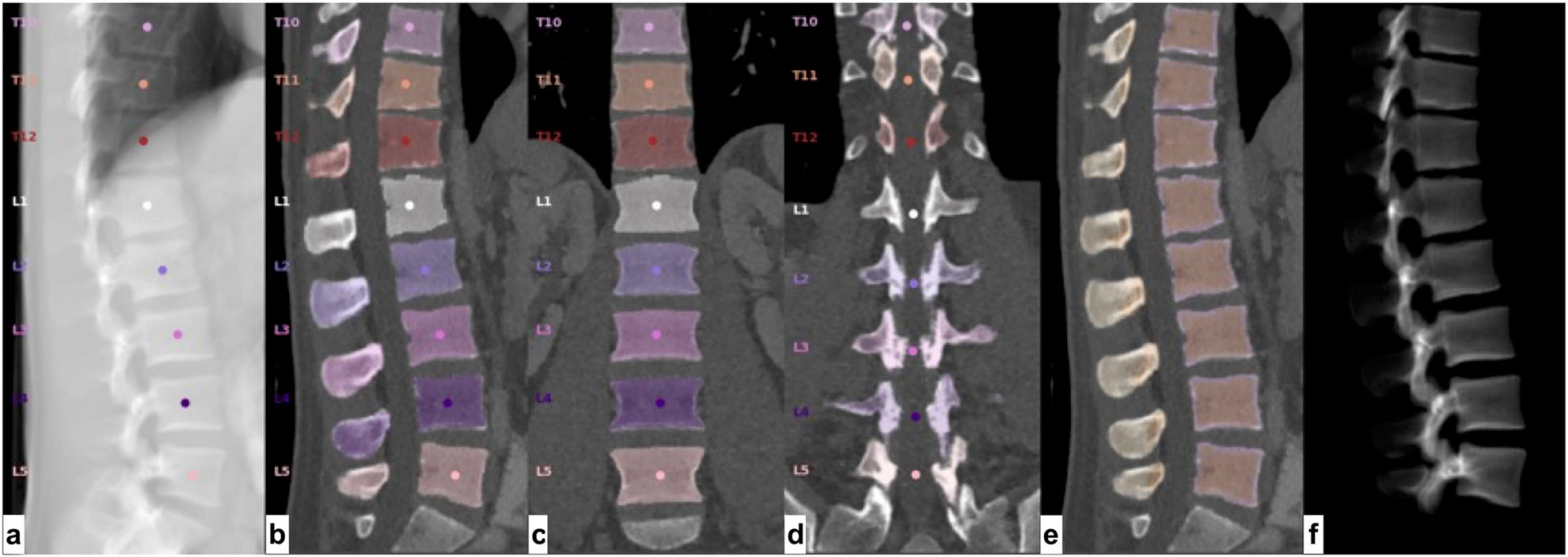
Steps of the automated segmentation by Bonescreen. **a** Vertebral body detection and labeling. Vertebral segmentation (**b**, sagittal view**; c**, coronal view), including posterior elements (**d**). **e** Identification of cortical and trabecular bone. **f** Three-dimensional model of segmented vertebrae

In all available reconstructions, automated spine detection, vertebral labelling, and trabecular compartment segmentation steps were performed automatically (SpineQ, version 1.0, Bonescreen GmbH, Munich, Germany, Fig. 1). HU-to-BMD calibration was performed automatically using linear conversion factors based on kVp and scanner type. Automated correction for the contrast media phase was performed using a two-dimensional DenseNET model [12]. Mean trabecular vBMD was calculated across the L1-4 lumbar vertebra (vBMD_L1-4_) for each observation. Vertebrae considered as unmeasurable according to the ACR criteria [15] were excluded from this mean on a per-patient basis. Specifically, vertebrae with fractures or Modic type 3 changes affecting > 25% of the vertebrae were excluded. For quality assurance, a neuroradiologist trained in spine imaging (P.P., 3 years of experience) reviewed vertebral body segmentation, automatic contrast media phase detection, and supervised in-/exclusion of vertebrae, documenting any manual corrections, if necessary.

### Group definitions

Acquisition parameters such as kVp, reconstruction kernel, and slice thickness, as well as scanner model, scan positioning, intravenous contrast media phase, and calibration status are well-known confounders of vBMD measurements [12, 16–19]. To quantify each factor’s impact on reproducibility, we defined a set of six groups with an expected increase in variance. Observations were assigned to groups based on the following criteria:

I. *Δ*_*recon*_: both measurements derived from a single acquisition but using different reconstruction kernels and / or slice thicknesses;
II. *Δ*_*contrast*_: measurements obtained from different contrast media phases, obtained in consecutive acquisitions during a single scan session;
III. *Δ*_*session*_: measurements derived from different scanning sessions at a single calibrated scanner, but both scans had the same contrast media phase;
IV. *Δ*_*scanner*_: measurements assessed in the same contrast media phase, but at two different calibrated scanners;
V. *Δ*_*all*_: measurements obtained from two different scanners, in different contrast media phases;
VI. _*scanner-agnostic*_: measurements obtained from datasets from two different scanners, at least one of which was not asynchronously calibrated, and only a kVp-specific, but scanner-independent, calibration was applied.

Groups I-V consisted of observations from asynchronously calibrated scanners only. Reconstruction kernels, slice thicknesses, and reconstruction planes were not controlled for and varied randomly as per acquisition protocol.

### Statistical analysis

All statistical analyses were performed using STATA software version 13.1 (StataCorp LLC, College Station, Texas, USA).

Absolute interscan differences in vBMD_L1-4_ were calculated by subtracting the vBMD_L1-4_ measurement 2 from the vBMD_L1-4_ measurement 1, in each observation, respectively. Relative interscan differences were calculated as percent gain or loss in vBMD_L1-4_ between measurement 1 and measurement 2, in all observations. Mean absolute vBMD differences and relative vBMD differences and respective standard deviations were calculated groupwise (I–IV).

Root mean square error (RMSE) and root mean square coefficients of variation (RMSCV) were calculated as measure of variance, for each group. To further investigate agreement of both measurements on single observation level, Bland Altman plots were created including 95% confidence intervals (95% CIs) of limits of agreement (mean difference × 1.96 standard deviation of the difference). The statistical significance of group differences in coefficients of variation was assessed using unifactorial ANOVA and Tukey post-hoc test.

Observations with poor agreement, defined as absolute difference exceeding the inner boundaries of the 95% CI limits of agreement, were retrospectively reviewed by an experienced neuroradiologist, specialized on spine imaging (J.S.K., 22 years of experience), and possible factors influencing vBMD measurements were recorded manually.

## Results

### Group statistics

**Fig. 2:**
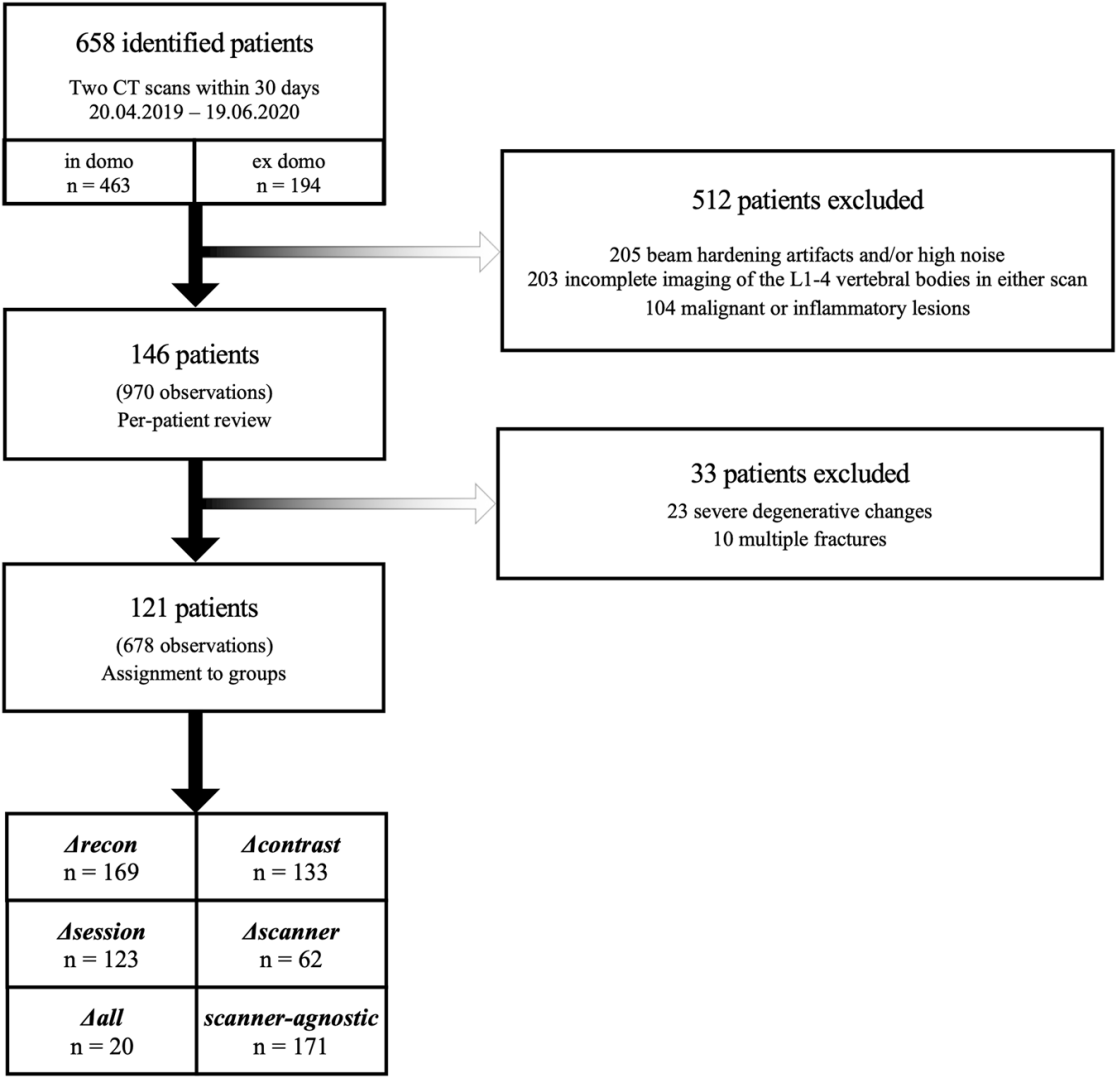
Flowchart depicting the inclusion process

Following the criteria for inclusion, 970 observations were derived from 146 patients (61 females, 41.8%), aged 63.4 ± 15.0 years (mean ± standard deviation), for a total of 292 patient scans total (146 × 2) (Fig. 2). After excluding observations without evaluable lumbar vertebrae, 679 observations were assigned to groups (Table 1). Across all observations, mean vBMD_L1-4_ was 173.4 mg calcium hydroxylapatite (CaHA) / cm^3^ (range 55.4–302.3) at baseline and 173.4 mg CaHA/cm^3^ (45.3–313.4) at follow-up. Slightly lower values were noted at measurement 2 compared to measurement 1: -0.0 ± 8.8 mg CaHA/cm^3^; -0.1%, *p* = 0.962. Observation numbers were greatest in the *Δ*_*recon*_ group and decreased through groups II–VI. Notably, the 133 observations assigned to *Δ*_*contrast*_ derived from 43 patients only, while 123 observations in the *Δ*_*session*_ group derived from 55 patients.

**Table 1:** Cohort demographics

Parameter		Unit	*Δ*_*recon*_	*Δ*_*contrast*_	*Δ*_*session*_	*Δ*_*scanner*_	*Δ*_*all*_	_*Scanner-agnostic*_
Patients		Number	72	43	55	31	10	23
Observations		Number	169	133	123	63	20	171
Sex^a^	Male	Number (%)	102 (60%)	90 (68%)	78 (63%)	37 (59%)	13 (65%)	110 (64%)
	Female	Number (%)	67 (40%)	43 (32%)	45 (37%)	26 (41%)	7 (35%)	61 (36%)
Age^a^ (years)		Mean ± standard deviation	60.0 ± 14.4	64.0 ± 13.0	59.2 ± 14.8	57.7 ± 15.3	65.7 ± 8.4	61.8 ± 13.5
Location	in-house / in-house	Number (%)	153 (91%)	116 (87%)	123 (100%)	63 (100%)	20 (100%)	22 (13%)
	out-of-house / in-house	Number (%)	0	0	0	0	0	149 (87%)
	out-of-house / out-of-house	Number (%)	16 (9%)	17 (13%)	0	0	0	0

^a^ Values based on a number of observations

### Reproducibility measurements

As expected, RMSCV values increased along the groups from a minimum of 1.3% in measurements obtained from identical scans (Δ_recon_) to a maximum of 5.9% in the *Δ*_*all*_ cohort (Table 2). The RMSCV increased significantly between Δ_recon_ and Δ_contrast_ (*p* < 0.001) and Δ_contrast_ and Δ_session_ (*p* < 0.001), while the increases between Δ_session_ and Δ_scanner_ as well as Δ_scanner_ and Δ_all_ were not statistically significant (*p* ≥ 0.770, respectively). The RMSCV difference between Δ_all_ and _scanner-agnostic_ did barely not reach statistical significance (*p* = 0.054). As reflected in the RMSCV, vBMD_L1-4_ values for both measurements in the Δ_recon_ group (*n* = 169) were extremely similar, with an average absolute difference of 0.1 ± 3.1 mg CaHA/cm^3^ and a relative difference of 0.1 ± 2.3%. Consequently, the group showed an overall low absolute error with an RMSE of 3.1 mg CaHA/cm^3^.

**Table 2:** Reproducibility of fully automated vBMD_L1-4_ measurements in each group

Group name	Group size	Measurement 1 vBMD_L1-4_ [mg CaHA/cm^3^]	Measurement 2 vBMD_L1-4_ [mg CaHA/cm^3^]	Absolute difference [mg CaHA/cm^3^]	Relative difference [%]	RMSE [mg CaHA/cm^3^]	RMSCV [%]
	*N*umber	*Mean ± standard deviation*	*Mean ± standard deviation*	*Mean ± standard deviation*	*Mean ± standard deviation*		
*Δ*_*recon*_^a^	169	175.3 ± 54.8	175.4 ± 54.7	0.1 ± 3.0	0.2 ± 2.1	3.0	1.3
*Δ*_*contrast*_^a^	133	171.2 ± 53.7	170.1 ± 55.3	-1.0 ± 6.4	-0.9 ± 4.5	6.1	2.9
*Δ*_*session*_^a^	123	160.1 ± 54.0	162.8 ± 58.9	2.7 ± 12.5	1.3 ± 7.4	11.0	4.7
*Δ*_*session_no-outliers*_^a^	111	156.4 ± 51.4	157.0 ± 53.6	0.6 ± 9.5	0.3 ± 5.9	9.0	4.1
*Δ*_*scanner*_^a^	63	164.1 ± 56.6	162.0 ± 58.3	-2.1 ± 8.9	-2.0 ± 7.3	8.7	4.9
*Δ*_*all*_^a^	20	139.2 ± 43.7	131.5 ± 45.9	-7.7 ± 8.0	-6.3 ± 6.7	7.6	5.9
_*Scanner-agnostic*_	171	190.8 ± 51.7	191.1 ± 49.3	0.4 ± 10.1	0.9 ± 5.9	10.1	3.7

^a^ Group contains asynchronously calibrated measurements only. *vBMD* Volumetric bone mineral density, *CaHA* Calcium hydroxylapatite, *RMSE* Root mean square error, *RMSCV* Root mean square coefficient of variation. *Δ*_*session_no-outliers*_ comprises the *Δ*_*session*_ group following exclusion of outliers, which exceeded the inner boundaries of the 95% confidence interval limit of agreement of the Bland Altman plot in Fig. 4

**Fig. 3:**
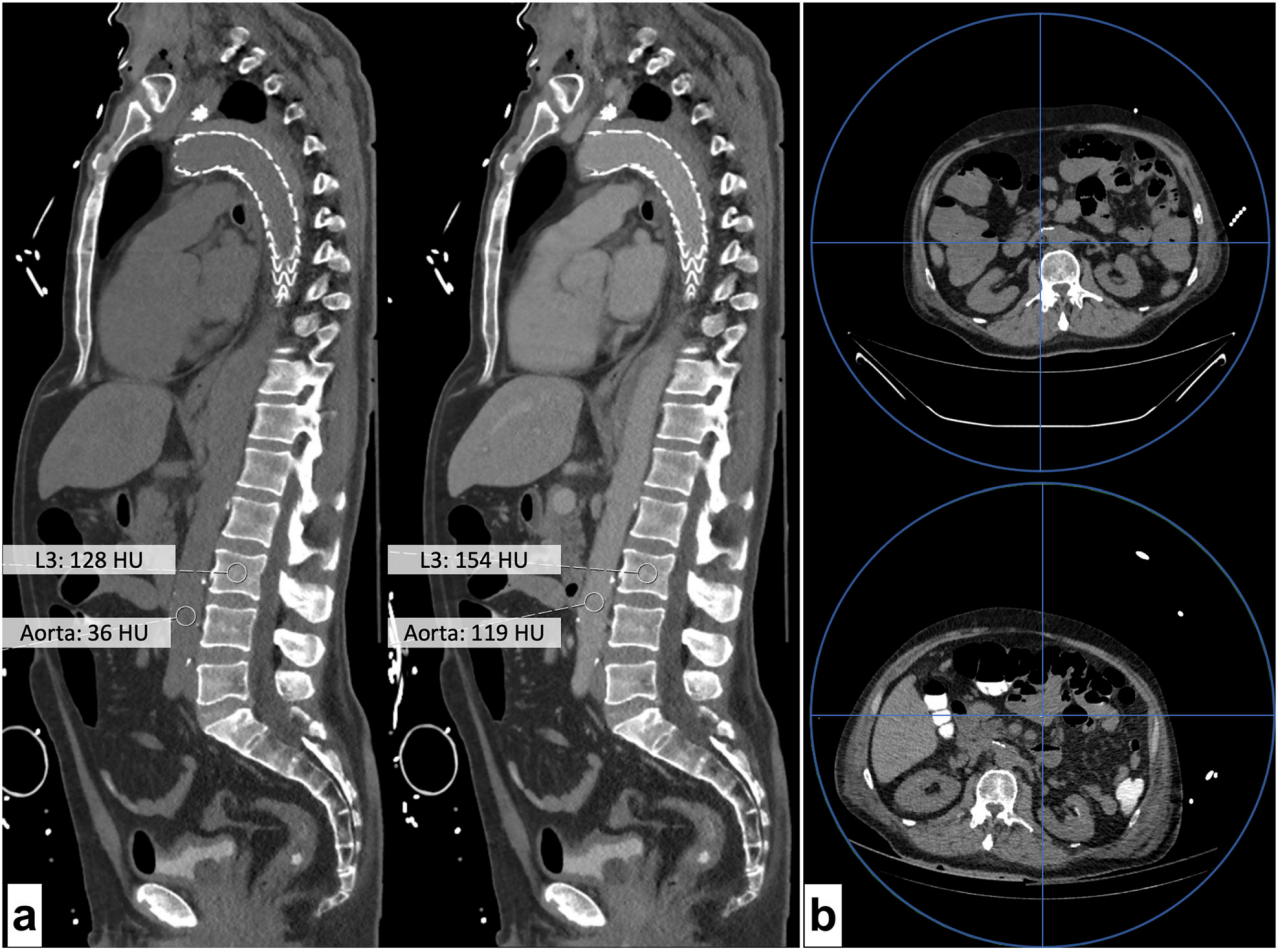
**a** Sagittal reformations of two thoracolumbar CT scans (left, unenhanced; right, 90 s after intravenous contrast media administration). Scans were obtained for search of an endoleak of the aortic prothesis (arrowhead). Annotations show HU of the abdominal aorta and the L3 vertebral body. Measured bone mineral density was 96 mg CaHA/cm^3^ in the unenhanced scan and 98 mg CaHA/cm^3^ in portal-venous phase. **b** Axial reformations of two unenhanced CT scans of a single patient obtained 20 days apart on the same scanner (Philips IQon). Images show cross-sections at the L3 level. Blue circles represent the maximum field of view. Blue lines intersect at the scanner center. The substantial difference in patient placement and distance between the lumbar spine and the scanner center between scans is evident and may explain substantial differences in bone mineral density measurements (top, 207.7 mg CaHA/cm^3^; bottom, 227.2 mg CaHA/cm^3^). Of note, the bottom scan was obtained following oral contrast administration

Observations containing measurements from different contrast media phases showed slightly greater dispersion, but overall, the absolute and relative errors remained low (*Δ*_*contrast*_ RMSE = 6.1 mg CaHA/cm^3^; RMSCV = 3.1%) (Fig. 3a). While measurements obtained from different scanning sessions (but the same scanner) had a greater absolute error of RMSE = 11.0 mg CaHA/cm^3^, the RMSCV remained below 5%. Measurements from different scanners showed greater differences (*Δ*_*scanner*_ absolute difference = -3.8 ± 10.0 mg CaHA/cm^3^; relative difference = -3.1 ± 7.8%), accompanied by increased errors (RMSE = 9.4 mg CaHA/cm^3^; RMSCV = 5.7%) (Fig. 3b). The relative error increased further in *Δ*_*all-observations*_, to 5.9%. Notably, measurements in *scanner-agnostic* scans showed acceptable absolute (2.0 ± 10.0 mg CaHA/cm^3^) and relative (2.0 ± 5.9%) differences, reflected in the mean absolute (RMSE = 9.6 mg CaHA/cm^3^) and relative error (RMSCV = 4.2%).

**Fig. 4:**
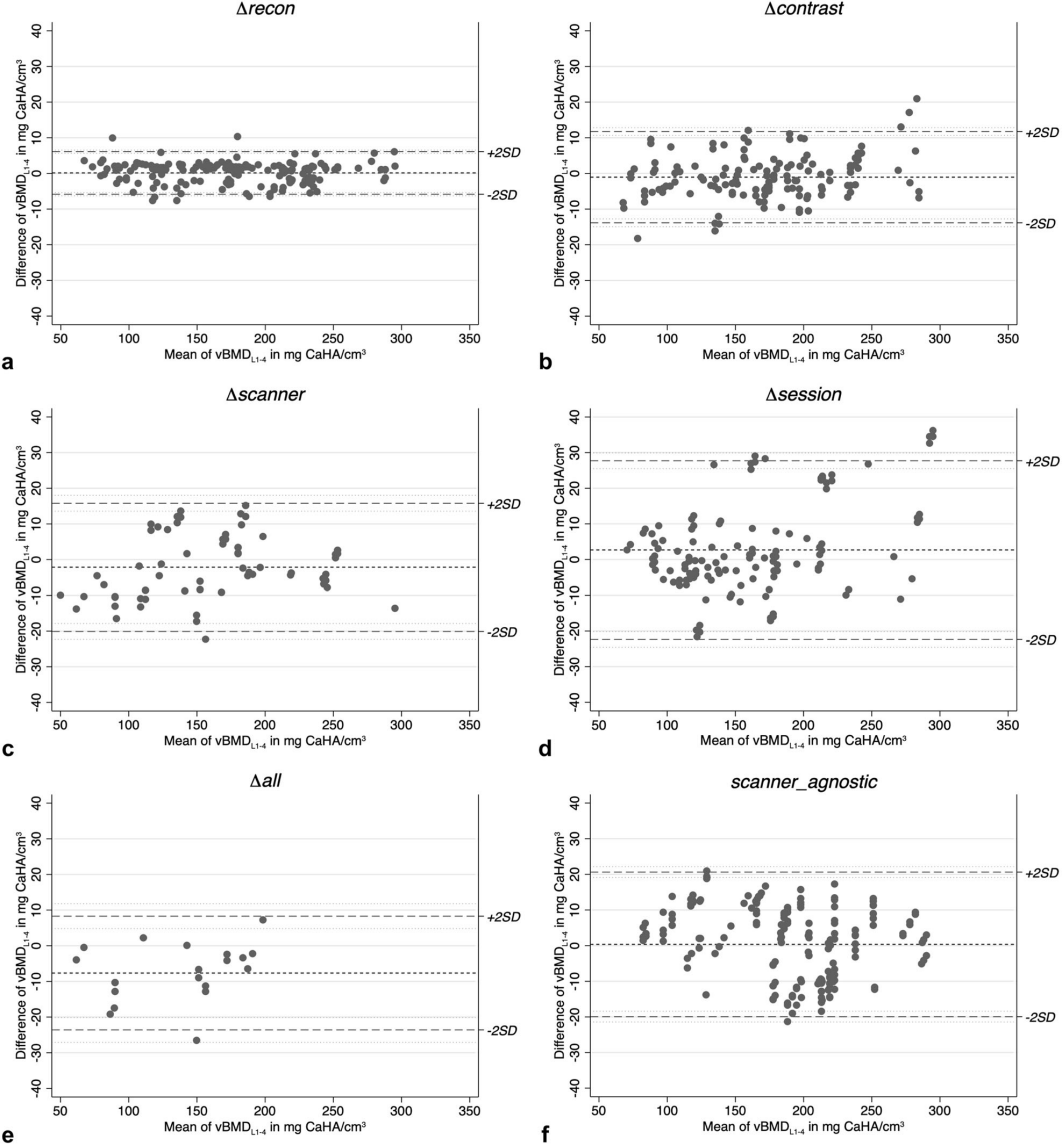
**a–f** Bland-Altman plots visualizing agreement of measurements on per-observation basis, in each group. For each observation (grey dots), the difference between measurement 1 and measurement 2 is plotted against the group mean. The group mean is indicated by the short-dashed line, while the long-dashed lines indicate the limits of agreement (± 2 standard deviations [SD]) (dotted lines)

**Fig. 5:**
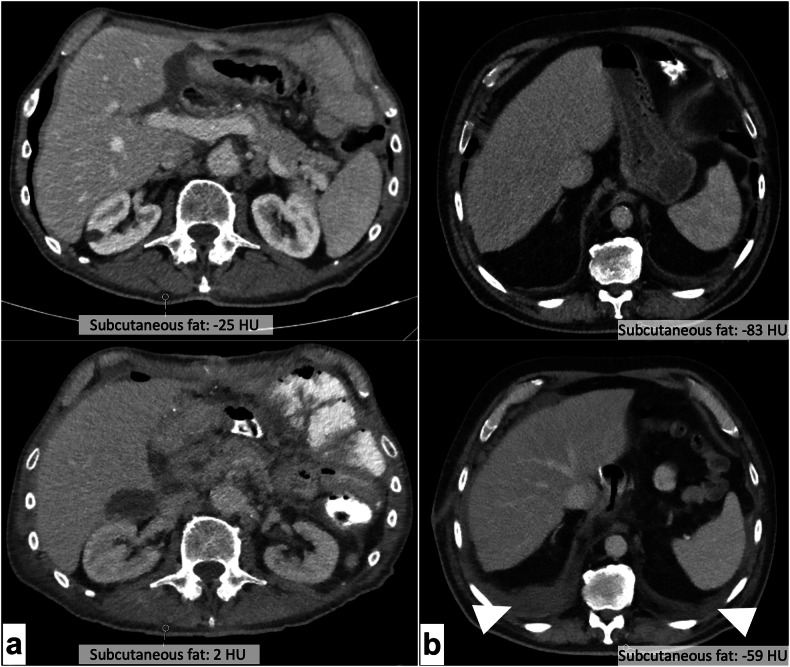
**a** Axial reformations of two scans of the same patient, obtained 16 days apart at the L2 level (top: baseline; bottom: follow-up). Derived bone mineral density measurements changed significantly between scans (baseline: 148.3 mg CaHA/cm^3^; follow-up: 175.1 mg CaHA/cm^3)^. Manual case review revealed that the patient suffered multiple intraabdominal abscesses between scans. Subcutaneous fat HU increased from -25 to +2 between scans as sign of hydropic decompensation. Also note the progressive mesenterial fluid injections and paracolic ascites. **b** Imaging at the L1 level revealed pleural effusion in the follow-up scan (bottom) of this patient, 19 days after baseline (top). The patient also showed an increase of subcutaneous fat attenuation from -83 HU at baseline to -59 HU at follow-up, co-occurring with an increase in measured bone mineral density (baseline 111.4 mg CaHA/cm^3^; follow-up: 133.1 mg CaHA/cm^3)^

Bland Altman plot analysis showed good agreement for all groups, except in *Δ*_*session*_. In this group, 12 observations exceeded the lower 95% CI of the upper limit of agreement (25.5 mg CaHA/cm^3^) or the upper 95% CI of the lower limit of agreement (-20.15 mg CaHA/cm^3^) (Fig. 4**)**. Manual review showed that all those patients were scanned twice within a short period due to severe illness with signs of hydropic decompensation, resulting in new or increasing pleural effusion (*n* = 11), anasarca (*n* = 10), mesenterial fluid injection or ascites (*n* = 5) and pulmonary septal thickening (*n* = 3). Two of the patients were intubated between baseline and follow-up and had received new abdominal drainages (Fig. 5). Exclusion of the identified outliers resulted in lower value dispersion and in improvement of absolute and relative precision errors (*Δ*_*session_no-outliers*_ *n* = 111; absolute difference = 0.6 ± 9.5; relative difference 0.3 ± 5.9; RMSE = 9.0; RMSCV = 4.1).

## Discussion

This study investigated the reproducibility of vBMD assessments from routine clinical CT scans using a fully automated convolutional neural network framework in various settings, including cross-scanner and cross-center settings. Reproducibility was excellent for all comparisons derived from a single scanning session. This demonstrates that neither the input convolution kernel nor slice orientation or thickness diminish reproducibility of this approach, and that the contrast media phase can be effectively corrected for. Precision errors for measurements derived from two different scanning sessions or different scanners were higher, but acceptable. While measurement errors may be due in part to changes in patient positioning, we also found evidence that measurement errors may also be driven up by short-term changes in tissue water content of severely ill patients and recommend introducing hydropic decompensation as a general exclusion criterion for quantitative CT measurements.

The *Δ*_*recon*_ group showed excellent reproducibility of vBMD_L1-4_ measurements with precision errors of approximately 1.5% or 3 mg CaHA/cm^3^. Similar precision has been shown for dual X-ray absorptiometry in repeated measurements without and with repositioning [20–22]. Since the two measurements in this group were derived from reformations created using different reformations of a single scan, the precision error is most likely attributable to slight differences in the segmentation process caused by the different reconstruction kernels, slice orientations and thicknesses, resulting in slightly different mean HU values or volume-of-interest placements by the convolutional Bonescreen neural network framework [10, 23]. Overall, the findings underline the previously reported robustness of the automated segmentation algorithm and HU-to-vBMD conversion following asynchronous calibration [10, 21, 24].

The influence of the contrast media phase on vBMD measurements is well known, especially in setups with external calibration, as the contrast medium augments the attenuation of vascularized body parts but does not influence the reference values obtained in the external phantom [8, 25, 26]. Therefore, a set of studies investigated correction methods for the contrast phase, and Rühling et al recently developed an automated model for contrast media phase detection and correction in a single-scanner setting [12, 25, 27, 28]. The study reported precision errors of 9.5 CaHA/cm^3^ in arterial and 4.0 mg CaHA/cm^3^ in portal-venous phase [12]. Contrast phase correction using phase-dependent correction factors performed similarly well in the current dataset, derived from various scanners, with absolute and relative precision errors of 6.1 mg CaHA/cm^3^ and 2.9% in the *Δ*_*contrast*_ group. Moreover, precision errors increased only slightly in the *Δ*_*all*_ group, compared to the *Δ*_*session*_ and *Δ*_*scanner*_ groups. This indicates that the implemented contrast media phase correction may work similarly well in cross-scanner comparisons. Modern advancements in CT, like spectral imaging, can further minimize the impact of intravenous contrast or other foreign materials on vBMD measurements and has been shown to yield promising results for vBMD measurements and in fracture prediction with good reproducibility [29–31]. While there is barely any use case for spectral CT in mass opportunistic osteoporosis screening due to the limited scanner availability, it may prove to be a pivotal advancement in osteoporosis diagnostics in the future.

Patient positioning and table height are known to severely impact attenuation values in multidetector CT due to x-ray field inhomogeneities and beam hardening effects being highly dependent on the scanner’s isocenter, and thereby also affect vBMD measurements [16–19]. This may in part explain the higher RMSCV and RMSE values in the *Δ*_*session*_, *Δ*_*scanner*,_ and *Δ*_*all*_ groups, as all observation pairs in these groups were obtained in different scanning sessions and to a certain extent, differences in patient positioning and table height can be expected between sessions. To encounter this problem, internal calibration has been proposed as an alternative calibration method. Internal calibration uses tissue-specific HU values, *e.g*., fat and muscle, to calculate a scan-specific conversion factor from HU to BMD [32]. While the method showed promising results in the past, studies have found that it is not superior to asynchronous calibration [27].

Across groups with patient repositioning, retrospective examination of cases with the greatest vBMD-variability revealed that the measurements were partially derived from severely ill patients from the intensive care units of our hospital, who had fluctuating levels of intra-abdominal as well as interstitial fluid and pleural effusion between scans. This bias is particularly difficult to correct for since hydration status in intensive care unit patients may substantially fluctuate daily. We regard this finding particularly important, as hydration status is not a reported confounder for quantitative CT measurements, which thus ought to be critically revised in severely ill patients. However, since our study was not specifically designed to investigate this topic, it warrants further investigation.

Data on cross-session reproducibility for asynchronous vBMD measurements is scarce, even more so for cross-scanner settings. Previous reports of reproducibility measures for asynchronous quantitative CT in single-scanner settings showed precision errors of 3−4 mg CaHA/cm^3^ or 2.2−3.7% [33, 34]. Sollmann et al compared opportunistically assessed vBMD from a set of six different scanners with QCT and documented different degrees of variation per scanner; an approach that seems somewhat comparable to cross-scanner results [11]. However, the authors did not measure cross-scanner reproducibility directly. With respect to the possible bias of the hydration status, precision errors across *Δ*_*session*_, *Δ*_*scanner*_ and *Δ*_*all*_ may be regarded as acceptable, with a maximum relative error of 5.9% in the *Δ*_*all*_ group and a maximum absolute error of 11 mg CaHA/cm^3^ in *Δ*_*session*_. _*Scanner-agnostic*_ observation pairs showed similar reproducibility to asynchronously calibrated measurements from different sessions, and _*scanner-agnostic*_ yielded better results than *Δ*_*all*_, and did only barely not reach statistical significance. In fact, both the RMSE of 10.1 mg CaHA/cm^3^ and the RMSCV of 3.7% were slightly lower in _*scanner-agnostic*_ compared to the *Δ*_*session*_ group and the RMSCV was markedly lower in _*scanner-agnostic*_ compared to *Δ*_*all*_. Both results suggest that scanner-specific phantom measurements may not necessarily be needed for asynchronous calibration if kVp-specific calibration factors are available for the scanner type.

We acknowledge some limitations of our study. First, we extended the generalizability of our results by including several scanners from outside of our institution. However, despite our efforts, we did not achieve an equal distribution across all main vendors. This may have an adverse impact on the external validity of the determined reproducibility. However, from our point of view, this issue can only be solved in multicentric studies, preferably with scanner-specific asynchronous calibration. Nonetheless, we demonstrated, that even *scanner-agnostic* kVp-based calibration yields acceptable reproducibility, regardless of the scanner combination. Second, cross-session reproducibility was limited, as we included many severely ill patients. It remains unclear, whether the observed increases in precision errors were attributable to patient positioning or caused by pathophysiological changes to the body composition like changes in the hydration status. Since this issue has not been reported on in the literature and this study was not designed to further investigate this finding, it necessitates further investigation. However, this problem appears to be difficult, as it seems to be inherent in the typical design for this type of study, because healthy individuals would rarely receive two thoracoabdominal CT scans within a single month.

To summarize, the automatic vBMD measurements by a convolutional neural network-based tool with asynchronous calibration and automated correction for the contrast media phase investigated in this study showed good reproducibility. The slightly lower precision in cross-session and cross-scanner settings may be related to patient positioning, but also short-term changes to the patients’ body compositions, necessitating further investigations. Notably, precision was similar in cross-session settings and the group without scanner-dedicated, asynchronous phantom-based calibration. Patient positioning and body composition may thus be of interest as major determinants of reproducibility for further studies.

## Data Availability

The datasets used and/or analyzed during the current study are available from the corresponding author on reasonable request.

## Abbreviations

BMD: Bone mineral density
CaHA: Calcium hydroxylapatite
CI: Confidence interval
RMSCV: Root mean square coefficient of variation
RMSE: Root mean square error
vBMD: volumetric BMD
